# So what do we really mean when we say that systems biology is *holistic*?

**DOI:** 10.1186/1752-0509-4-22

**Published:** 2010-03-12

**Authors:** Derek Gatherer

**Affiliations:** 1 MRC Virology Unit, Institute of Virology, University of Glasgow, Church Street, Glasgow G11 5JR, UK

## Abstract

**Background:**

An old debate has undergone a resurgence in systems biology: that of *reductionism *versus *holism*. At least 35 articles in the systems biology literature since 2003 have touched on this issue. The histories of holism and reductionism in the philosophy of biology are reviewed, and the current debate in systems biology is placed in context.

**Results:**

Inter-theoretic reductionism in the strict sense envisaged by its creators from the 1930s to the 1960s is largely impractical in biology, and was effectively abandoned by the early 1970s in favour of a more piecemeal approach using individual reductive explanations. Classical holism was a stillborn theory of the 1920s, but the term survived in several fields as a loose umbrella designation for various kinds of anti-reductionism which often differ markedly. Several of these different anti-reductionisms are on display in the holistic rhetoric of the recent systems biology literature. This debate also coincides with a time when interesting arguments are being proposed within the philosophy of biology for a new kind of reductionism.

**Conclusions:**

Engaging more deeply with these issues should sharpen our ideas concerning the philosophy of systems biology and its future best methodology. As with previous decisive moments in the history of biology, only those theories that immediately suggest relatively easy experiments will be winners.

## Introduction

An old debate has made a surprising, or perhaps not so surprising, reappearance in systems biology: that of *reductionism *versus *holism*. Surprising: in the sense that the mainstream of molecular biology has for at least 30 years been relatively uninterested in the matter, content with a compromise that allows lab work to proceed without too much philosophical agonizing. Unsurprising: in that general systems theory has churned with the debate for nearly 75 years. Once systems theory met molecular biology, it was probably only a matter of time before the controversy went pandemic. At least 35 articles in the systems biology literature from 2003 to the early part of 2009 touched on this issue [1-35]. However, much confusion is apparent in the variation in what is understood by the term *holistic*, and also from overstatements as to the degree that "traditional" molecular biology is truly reductionist. Some of the systems biology articles give the impression that this is the first time this argument has arisen, but we have been here before on several occasions. The reductionist-holist debate emerged in biology in the 1920s out of the earlier disputes between *mechanists *and *vitalists*, and between *neo-Darwinians *and *neo-Lamarckians*, although it should not be thought that all the corresponding terms on the left or right sides of each pair are in any way necessarily equivalent. One would not expect any aspiring modern holist within the systems biology community to be a vitalist or a neo-Lamarckian. Nevertheless, broadly speaking, it is possible to see general continuities that allow one to posit what might be called mechanist-Darwinian-reductionist and vitalist-Lamarckian-holist lineages, respectively, and an understanding of how these debates unfolded serves to illuminate how any modern reductionist-holist conversation within systems biology may develop. Those systems biologists who see themselves as holists need to know what kind of holists they are. Then they can be more explicit about what aspects of reductionism they are opposed to. Many holistically inclined systems biologists seem to be arguing against a classical and now rather outdated version of reductionism, designed for physics and never really implemented in molecular biology, and are apparently unaware of the new developments in the philosophy of biology, what one might call the neo-reductionism, of the last 20 years. Bringing systems biology face to face with the latest versions of reductionism should be the next step in the process. First, some of the previous rounds in the contest are reviewed.

## The Death of Vitalism

Vitalism is the belief that some special life-force (the *hormic schema*, *sentiment intérieur, élan vital *or *entelechy *depending on the author) is necessary to differentiate the living from the inanimate. For vitalists, no matter how much detail is understood about the physical and mechanistic nature of living things, no matter how much was known in the 19^th ^and early 20^th ^centuries about their anatomy, physiology and biochemistry, or since the mid-20^th ^century about their molecular biology and genetics, this knowledge can never explain why they are alive. This, of course, is a position with very deep religious roots. Reference to *vital spirits *as animatory factors in living matter was current among the biologists of the 17^th ^century, and there was an active anti-mechanistic movement among the group of 17^th ^century philosophers and liberal theologians known as the Cambridge Platonists [36], although *vitalism *was only first coined as a phrase by Paul-Joseph Barthez as late as 1778 [37].

Just as vitalism had deep theological roots, its great rival *mechanism *was the product of many centuries of practical problem solving with engineering devices, long predating science, but the integration of the device-centred world of the artist-engineer with the mathematico-theoretical one of the scientist-scholar was not commonplace until the time of Kepler and Galileo in the late 16^th ^and early 17^th ^centuries [36]. Modern writers often point to Galileo's younger contemporary René Descartes as the man who first synthesized these influences into a mechanistic theory of biology, although Cartesian specialists are often unsure if Descartes can really be interpreted in that way [38]. However, many of Descartes's followers certainly held this view - the title of Julian Offray de La Mettrie's *L'Homme Machine *of 1748 speaks for itself.

The philosophical basis for vitalism in its most recent form is often taken to be the work of Henri Bergson [39], and its last great scientific exponent was the embryologist Hans Driesch [40]. Driesch's work is still often studied at second-hand today by way of contrast with his mechanist rival Wilhelm Roux, who together with Driesch virtually created the modern experimental science of developmental biology out of the descriptive embryological anatomy of the 19^th ^century. Roux was more a laboratory man than a philosopher, so the main defence of the principles of mechanism, that all living processes "can be unequivocally explained in physicochemical terms" [quoted in [41], p.430] fell to Jacques Loeb [42]. That Loeb was a biochemist, rather than a developmental biologist like Roux, was no accident, since the discovery of cell-free fermentation by Eduard Buchner in 1897 is regarded as both one of the foundations of modern biochemistry and also a major experimental nail in the coffin of vitalism. By the 1920s, vitalism had been almost completely abandoned, not just because it had failed to convince practising biologists on a theoretical level but also on account of its inability to provide a basis for any experimental research programme, despite some interesting efforts in embryology by Driesch. Mechanism, by contrast, suggested numerous avenues of experimental analysis. Vitalism in its death throes was largely constituted as a denial of the possibilities of progress in understanding biological systems through mechanistic methods and this was a difficult position to hold when experimental progress was so obvious. Mechanism won the philosophical debate, as far as any philosophical debate can ever be said to be won, but more importantly for everyday science, it won on the practical level.

In the aftermath of Darwin's triumphant bicentenary, it is easy to forget that his centenary year and the two decades following it were difficult times for Darwinians. The problems of reconciling Darwin's gradualist view of evolution with the saltationist or macro-mutationist views common among the early Mendelian geneticists had given rise to a climate where the theories of Darwin's predecessor Lamarck, or a mutated form of them known as *neo-Lamarckism *which emphasised the inheritance of traits developed during the lifetime of the organism through interaction with its environment, were once again resurgent. *Evolution in the Light of Modern Knowledge *[43], a multi-author volume published in 1925 and typical of its time, contains neo-Lamarckian contributions from psychologist Conwy Lloyd Morgan, botanist E.O. Bower and zoologist Ernest MacBride, all leading representatives of their disciplines. There is not a single article that is recognizably Darwinian to modern eyes, and the only references to Darwin are attempts to co-opt Darwin's later theories as a kind of early neo-Lamarckism. Lloyd Morgan retains a lasting influence within the reductionist-holist debate via his development of the antireductionist concept of *emergent evolution*, still used today [44]. However, just as the mechanists had gained the upper hand over the vitalists by a combination of theoretical and practical advantages, so did this final flowering of Lamarckism perish at the hands of neo-Darwinism by the 1930s. The neo-Darwinian synthesis, pioneered by R.A. Fisher and others, had the theoretical advantage over Lamarckism of its reconciliation of Mendelian genetics with evolutionary gradualism, whereas the neo-Lamarckians remained unsure how to integrate Lamarck with Mendel. MacBride, for instance, thought Mendelian genetics to be of no evolutionary relevance. Neo-Darwinism also had the experimental advantage of proposing an immense raft of relatively quick and simple experiments, which soon became the basis of the disciplines of ecological and population genetics. The experiments of Lamarckism, by contrast, often involving behavioural or anatomical manipulations on laboratory animals, were long, laborious and difficult to interpret conclusively, as in the famous case of Paul Kammerer's midwife toads [45]. Since the last of the vitalists also tended to be neo-Lamarckians in their evolutionary theories, the defeat of Lamarckism was also the final straw for what remained of vitalism. Once again, the victory was won in the laboratory rather than in the theoretician's study.

## The Short Life of Classical Holism and the Birth of Inter-Theoretic Reductionism

It was into this environment that Jan Smuts first launched his concept of holism [46]. From the Greek õλoζ (a whole), Smuts' wholes were the basis of a philosophy not dissimilar to Lloyd Morgan's emergent evolution. For Smuts, the entire universe was based on an innate tendency for stable wholes to form from parts. This tendency was taken to occur at all levels from the atomic through the biological to the psychological. Whereas the *élan vital *of the vitalists was simply a factor that operated in living things to differentiate the animate from the inanimate, Smuts' *vera causa *operated in all matter, driving evolution ever upwards to larger and more complex new levels. As in the anti-Darwinian *orthogenetic *theories of evolution, the first of which was advanced by St. George Jackson Mivart in the 19^th ^century [47, pp.55-56], an upward, diversifying force is inherent in the material in question rather than being a consequence of the mechanistic interaction of parts, but Smuts wanted his principle to apply to the entire universe and not just biological organisms. "All the problems of the universe, not only those of matter and life, but also and especially those of mind and personality, which determine human nature and destiny, can in the last resort only be resolved - in so far as they are humanly soluble - by reference to the fundamental concept of Holism" [[46], pp. 320-321]. Not far removed from the *panpsychism *of the 17^th ^century philosopher Spinoza, which also advocated purposive properties in all matter, Smuts's holism can therefore be seen to be an attempt to salvage something from the debris of the vitalist-Lamarckian wreckage. Like his neo-Lamarckian biologist contemporary Ernest MacBride, Smuts did not regard Mendelian genetics as having any evolutionary importance, and his orthogenetic theory of evolution also relegated natural selection to a subsidiary role. Within ten years of the publication of Smuts' book, his theory was effectively obsolete in biology. Nevertheless, the term holism lived on and began to resurface in debates rather distant from the metaphysical questions with which Smuts had concerned himself.

Throughout his writing, it is clear that Smuts, although not strictly a vitalist, saw his main enemy as mechanism, rather than reductionism. The reductionist-holist dichotomy had to wait until reductionism was itself mature. Some modern authors have traced reductionism, like mechanism, back to Descartes [48,49] or even to ancient Greece [50,51], but its most ambitious modern form only dates from Smuts's contemporaries, the *Wiener Kreis *or Vienna Circle, a group of philosophers first organized in 1922, although some of the members had been associated before the First World War. The Vienna Circle adhered to *positivism*, a system that originated in the work of 19^th ^century philosopher Auguste Comte, and which was characterised by rejection of metaphysics and insistence on empirical data as the only true foundation of knowledge. Naturally, this led them to reject vitalism and embrace the mechanism of the experimentalists, attempting to construct a framework for a *unified science *built hierarchically on the foundations of physics: "*the whole of Science becomes Physics*... every scientific statement can be interpreted, in principle, as a physical statement" (capitals and italics in original) [[52], pp. 98-99], or as Thomas Nagel has expressed it: "Reductionism is the idea... that physics is the theory of everything" [[51], p.3] To this end, the Vienna Circle developed a rigorous method, using the grammar of mathematical logic, for the reduction of higher-level theories to more fundamental ones: the process of *inter-theoretic reduction*. Once reduced, the higher-level theory then becomes essentially unnecessary, except possibly as a kind of shorthand. Often referred to as layered-model reductionism by modern philosophers of science, this theory envisaged chemistry as based on physics, biology based on chemistry, and the human sciences (psychology, sociology etc) as based on biology. The Vienna Circle was broken up by the rise of the Nazis, and their other ideas on the philosophy of science were largely superseded by those of Karl Popper (they were *verificationists *whereas Popper was a *falsificationist*) who, thanks to the efforts of Peter Medawar [53,54] among others, became the mascot philosopher of molecular biology. Nevertheless, inter-theoretic reduction achieved its mature form in the early 1960s in the work of the Vienna Circle's disciples such as Ernest Nagel [41], and its logical form can be summarised as follows [55]:

If a reducing theory ***T ***is taken to reduce theory ***T'***, then

a) all laws of ***T' ***must be derivable from ***T***

b) all the theoretical vocabulary of ***T' ***must be re-expressible in terms of ***T***

So for instance, the term *temperature of a gas *in thermodynamics would be reducible to *mean kinetic energy *of the gas molecules in statistical mechanics and *gas pressure *would be reducible to *mean molecular density *of the gas molecules. By a steady accumulation of equivalences of this sort, thermodynamics would eventually be reduced to statistical mechanics. The vocabulary of thermodynamics would then become a kind of shorthand for concepts that could be expressed more completely, if rather more verbosely, in the language of statistical mechanics. Likewise, any reduction of Mendelian genetics to molecular biology would require re-expression of the terms of the former into those of the latter. *Gene *might thus be re-expressed as *stretch of DNA coding for a protein or functional RNA along with contiguous regulatory DNA*, and so on. Of course, there is considerable room for manoeuvre in this kind of definition: anyone who has been an undergraduate in genetics or molecular biology since the 1970s will be familiar with the workhorse examination question, "What is a gene?" Generations of students are thereby invited to do a little inter-theoretic reduction in the spirit of the Vienna Circle. Examinations aside, little attempt was actually made to apply this framework to real cases within the biological sciences but, due to popularizations by Francis Crick ("The ultimate aim of the modern movement in biology is in fact to explain *all *biology in terms of physics and chemistry" [[56], p.10, italics in original]) and Jacques Monod [50] in the 1960s and 1970s, it became taken for granted within molecular biology. Like Richard Dawkins' "unbelieving Anglicans" [57], molecular biologists had a philosophy they could comfortably pretend to agree on, that provided an ideal towards which they could reasonably strive, without any interference in the basics of decent daily activity.

Even assuming that both aspects of the inter-theoretic reduction were completed, with adequate re-expression of both vocabulary and laws, the completed reduction is still vulnerable to change in either or both of the reduced and reducing theories. If some aspect of biology is regarded as reduced to physics or at least chemistry, the assumption is made that the underlying chemistry is correct. Should the relevant parts of chemistry be disproved, the reductional chain will be broken, and the biology will require to be re-reduced to the new physics or chemistry. Previous satisfactory reductions may suddenly become invalid in this way, and reduction must always therefore be considered to be provisional. However, just as a previous reductive chain may be broken by changes in the underlying theory, so may reduction become possible where before it was not [58,59]. Anti-reductionist declarations must always be provisional too. Ernest Nagel interestingly points out that chemistry is only reducible to post-1925 physics, and thermodynamics is only reducible to post-1866 statistical mechanics. In both cases it was advances in the reducing theory that enabled the reduction [60].

## Modern Holism: 1) Epistemological Antireductionism

Meanwhile, despite the failure of Smuts to find any disciples among practising biologists, his label of holism was adopted by those opposed, for often quite divergent reasons, to the advance of reductionism [48,61-65]. Some of the post-war holists saw reductionism as a rather brutalizing tendency to describe all complex phenomena as "nothing but" the interaction of molecules, which they ridiculed as *nothing-buttery *[66]. The psychologist Abraham Maslow apparently even considered a reductionist attitude to be a mild form of psychoneurosis [quoted in [67], p.7]. Reductionists in return accused holists of *morethanism*, an allegedly emotional insistence that complex phenomena are necessarily "more than" the sum of their parts. More recently an intermediate position has developed, which believes in the essential value of reductionism but decries its excessive implementation as the sin of *greedy reductionism*. By the same token, it is also possible to be a *greedy holist *[68].

However, descriptions of these disputes often make the assumption that each of these parties represents an agreed philosophical position, which is rarely the case. Thomas Nagel has presented a taxonomy of modern anti-reductionism(s), which he divided in the first instance into two kinds: epistemological and ontological [51]. Epistemology being the branch of philosophy dealing with what can be known and how we know it, *epistemological antireductionism *is the recognition that some phenomena are too complex to be comprehended by human, or even computer, intelligence. Nagel's examples include a performance by a pianist - a description of every event in the performance at the quantum physical level, or possibly even at the muscular physiological level, would be overwhelmingly complex. The epistemological antireductionist is a holist because complete reductionism is *technically *impossible. For a systems biologist, sheer complexity, one might say *irreducible complexity *[13,69], can prevent the exhaustive analysis of a network of any interesting size. Quite how limiting our epistemological shortcomings can be is not fully appreciated, especially by those who advocate a *singularitarian *vision of a future world where "everything" is known [70]. To take a simple example, "Moore's Law" [71] observes that available processing power doubles every 2 years or so, and beyond the physical limits of present processors there is the promise of quantum computing. The "ultimate laptop", a quantum computing machine of approximate size and weight to a modern laptop performing at physically possible limits [72], would run at 10^51 ^FLOPS (c.f. 10^10 ^FLOPS for a current processor), based on the maximum information carrying capacity of 1 kg of matter, known as "Bremermann's Limit" [73]. 10^40 ^FLOPS will be the effective ultimate limit, for various technical reasons. This kind of mind-boggling computing power, possibly or even probably available within the next century, is the basis of singularitarian confidence. However, the biological systems are even more mind-boggling in their complexity. For instance, 193 genes are known to be involved in spore formation and degradation in *Bacillus *[74]. If we wish to make a start on simulation of the contribution of these 193 genes to the final phenotype, we might represent this network as 193 binary nodes, in the simplest possible star topology, i.e. all genes are either on or off and all have an equal weight in determining the state of the pathway and there are no interactions between genes. This is of course already a considerable simplification for the sake of easier modelling. An exhaustive exploration of such a state space of 193 inputs requires 2^193 ^or about 10^58 ^operations. A current processor requires about 10^48 ^seconds for this, or about 4 × 10^40 ^years. The ultimate laptop manages in just under 4 × 10^10 ^years, or slightly under 3 times the age of the universe. It is therefore clear that even this simplified 193 node model will never be fully computed on a single processor. Parallelisation has often been held up as a solution to such combinatorial explosions. If one were to construct an ultimate grid of ultimate laptops, the current limit would be 10^12 ^nodes, for various technical reasons. This would bring the total time down to practical levels, although the sheer energy demands of the system might present other restrictions. One alternative is simply to study only tractable problems. On a single current 10^10 ^FLOPS processor over one month, one could exhaustively explore a state space of 2.7 × 10^16 ^possibilities. A binary star-topology network of 54 genes is thus at the limit of brute force tractability. Even with the ultimate laptop, the corresponding figure is 154 genes. It is a sobering thought that a transition from present day computing capacity to ultimate computing capacity will only increase the tractable size of a binary star network by *a mere 3-fold *[69].

Expressions of antireductionism in one of systems biology's precursors, the classic systems theory literature, are also often of this epistemological kind. Ervin László, for instance, uses an example of understanding traffic accidents on the 4^th ^of July [75]. At the level of the individual drivers and cars (which is not even at the physical level), there would be many millions of data points, and complete uncertainty about how to use the various driver abilities, journey times and lengths, vehicle brake qualities and so on to derive a prediction of the number of accidents on any particular 4^th ^of July. A better understanding might be achieved by accessing statistics on previous accident blackspots, the weather in these locations and the alcohol consumption of drivers during the day. László does not deny the principle of reductionism, but merely questions its feasibility and usefulness. This is the mildest form of holism, but among the prominent holists of the postwar era many go beyond the epistemological argument and adopt ontological antireductionism.

## Modern Holism: 2) Ontological Antireductionisms, Constitutive and Explanatory

Nagel's second category is *ontological antireductionism*. Ontology, the study of what objects exist, or what is correctly defined as an object for study, is more familiar term for biologists, having become incorporated extensively into systems biology and bioinformatics. Ontological antireductionism is the argument that there are certain things, entities or laws, that reductionism can never capture, even if we transcended the epistemological limitations. Regardless of any hypothetical complete description of the pianist's performance it would fail to answer some questions one might have about the performance. Likewise, the ontological antireductionist would maintain that even if we could transcend Bremermann's Limit and derive a complete description of our irreducibly complex network, there would still be things that would remain unknown about it. Nagel then divides ontological antireductionism into two kinds, constitutive and descriptive, which give different reasons for this failure. *Constitutive ontological antireductionism *declares that there are fundamental things involved in the pianist's performance other than the particles described in the complete quantum mechanical description. One might insist that there would be some spiritual or musical aspect of the pianist's performance that could never be explained by the reduction to physiology or physics. Whatever the merits of such an argument in music theory, in our systems biology network example this would be tantamount to vitalism, and so can be effectively neglected.

*Explanatory ontological antireductionism*, by contrast, accepts that the physical description of the pianist's performance is the full description, but holds that there are emergent laws pertaining to that description that are not derivable from the laws governing the ultimate physical level, or aspects of a complete description that are not captured at the physical level. There is usually the implicit assumption that such emergent laws are in principle non-reducible, not that we merely fail to reduce them based on our current knowledge. Nagel's example of how such an emergent law could arise requires us to differentiate between physical states or events and facts about the world. One might acknowledge that every physical event has an explanation in terms of physics, but that a fact can have many physical descriptions that satisfy it. In the piano recital example, there are innumerable physical descriptions that would fit the pianist striking a certain key at a certain time. It is therefore wrong to insist that a higher level fact reduces to *a *physical explanation, as many physical explanations would be compatible with it. As macro-states of a system correspond to numerous micro-states, there is a certain buffering against changes at the micro-level. This has been termed *variostability *[76], *order above inhomogeneity *[77] or *cohesion *[78], and under such circumstances reduction may be deemed to be both "pointless and misleading" [78]. Likewise, given this apparent freedom of the higher-level property from total dependence on lower-level properties, higher-level laws are not derivable from lower level laws. Walter Elsasser coined the term *biotonic laws *for such laws applicable to biology which were compatible with the laws of physics, but not deducible from them [79]. Under such circumstance, ontological antireductionists often suggest that the higher-level systems may start to exert an effect on the lower level ones, a *top-down causation *or *macrodeterminism *[63,80] that reverses the usual *bottom-up causation *of the reductionist. Michael Polanyi extended antireductionism beyond biology to any complex objects such as motor cars [[81], pp. 124-142]. For Polanyi, both biological organisms and mechanisms of human construction work in accordance with physical laws but this does not therefore mean that they are fully comprehensible in terms of them. Polanyi stressed that organisms, and indeed complex mechanical objects like motor cars, have *pathologies *in the way that inanimate open systems, such as thunderstorms, do not. This is not an argument about complexity; the thunderstorm is itself highly complex, indeed chaotic in the formal mathematical sense. There are many ways that an organism can go wrong, can be sick, fail to eat, defend itself, reproduce or even die. Likewise, a motor car can break down or fail to start. By contrast hydrochloric acid can never fail to dissolve zinc, and a thunderstorm can never fail to shed large quantities of rain. Polanyi used the word *achievement *to describe this property. We have to consider organisms as achieving or failing in certain goals, otherwise we cannot comprehend what organisms are doing at any time. Polanyi is often regarded as being as antireductionist as it is possible to be, but his concept of pathologies in biological and mechanical devices is a strong argument for mechanism. Organisms and mechanisms are differentiated from chaotic natural systems like thunderstorms. Claiming that mechanisms are simplistic organisms is merely the converse of the standard mechanistic argument that organisms are just highly complex mechanisms.

## A Bestiary of Holisms in System Biology

While these discussions were ongoing, practical molecular biology carried on regardless for some 50 years, reasonably comfortable in its tentative reductionism. Experimental success caused holistic objections to fade away. Single gene analyses gradually shifted towards large mutational screens and complete genome maps, then to whole genome sequences and exhaustive genomics and proteomics expression data. Almost imperceptibly, systems biology emerged out of traditional molecular biology, and with this shift the reductionism-holism debate has resurfaced, with many recent bold declarations that systems biology is a more holistic approach to biology than traditional molecular biology [15,16,23,28,30,34,35], or arguments that traditional molecular biology represents a greedy reductionist approach (to some authors a naïvely reductionist one) that either requires extensive complementation from, or maybe even replacement by, systems biology [12,13,19,22,24,33]. In some cases, these comments have come from research areas that already regard themselves as more holistic than molecular biology and which identify a kindred spirit in systems biology. Examples include ethnopharmacology [15] or Chinese traditional medicine [16]. Some papers have emphasised top-down causation as a property of systems [4,10,14,21,23,31]. Others [1,20,21,23,29,31] have even suggested that systems biology is the beginning of a *paradigm shift*, a fundamental change in the way we do biology, comparable to past paradigm shifts such as the 17th century transition from Ptolemaic to Copernican astronomy or the early 20th century transition from classical to modern physics [82], or that we are witnessing the death of molecular biology as we know it [32].

Not everybody has agreed. Some think that such declarations of holism are wrong because systems biology is actually not holistic enough [2,3,7,18,25] and so far is really just old-fashioned molecular biology writ large: "a euphemism for gathering ever more details on an ever larger scale" [2]. A rather smaller group of dissenters take the opposite line entirely, disagreeing with holism on principle, and arguing for an even more reductionist approach [9,11]. For instance, it has been argued, looking at systems biology from the perspective of chemical process engineering, that systems biology does not suffer from data overload, but in fact is rather data-poor in those quantitative, precise measurements that are the most necessary for accurate network simulation [11]. Others have expressed various degrees of skepticism concerning the paradigm shift claims [26,83]

Nevertheless, nobody nowadays, at least in the systems biology literature, is advocating the original holism of Jan Smuts. Some authors, although enthusiastic about the anti-reductionism they see in systems biology, find that connotations with the work of Smuts make them uneasy with the use of the term holism [23]. Its original formulation having been dropped, it is not now entirely clear what a modern holistic systems biology is or would be. This is reflected in the variety of ways in which the term is currently used. Some see it simply as a change in emphasis in the size of the units of analysis - if we are studying whole pathways rather than individual genes, that must be (w)holism. Others see a problem of circularity in explanation - cellular systems behaviour cannot be explained in terms of gene action as the gene action is itself part of that system. These kinds of holisms are really just general statements of intent. Just as traditional molecular biologists were reductionists without being sure of what reductionism entailed, so are these systems biologists similarly holists by declaration rather than practice. Where more detailed arguments for holism occur in the systems biology literature, they are often ontological explanatory antireductionist [4,5,10,12,14,17,21,23,31], but it is also possible to see some that are of the milder epistemological antireductionist kind [9,28]. The choice of the word holism by systems biologists, rather than any of its more common modern synonyms *organicism*, *emergentism *or *coherentism*, is itself intriguing but there does not appear to be any obvious explanation. It may be that holism is largely found within the theoretical biology literature, whereas emergentism and coherentism are more used by philosophers. Organicism perhaps implies the properties of a whole organism which is not usually the level under discussion in systems biology.

## The Death of Inter-Theoretic Reductionism in Biology

We may not know exactly what modern holism in systems biology is - although we can perhaps generalise that it is usually explanatory ontological antireductionism with some tendencies to epistemological antireductionism - but we do know that it is against reductionism. That might be the end of the discussion, were it not for the fact that it is not entirely clear if we know what reductionism is either. Scientists can see vaguely what it means to reduce biology to chemistry, to say that biological phenomena are explicable in terms of chemical reactions, and can often provide specific and convincing examples, but doing inter-theory reduction in its rigorous form is very difficult in biology. Following on from the framework of Ernest Nagel described above, in the late 1960s and early 1970s some attempts were made by Kenneth Schaffner and David Hull at an inter-theoretic reduction of Mendelian genetics to molecular genetics, with mixed results [84,85]. For instance, there is no term in molecular biology that can capture everything that is implied by the term *gene *in classical genetics. Molecular biologists know that genes are made of DNA, but each gene is unique in terms of how that DNA is constituted into that particular gene. Reduction is challenging enough for objects in the two theories but for processes, such as segregation or gene silencing, the complexities are even greater. For instance, Philip Kitcher has pointed out that the independent assortment of genes at meiosis can be explained simply in terms of a set of rules for moving objects. Even if those objects are not actual chromosomes but simulations, e.g. beanbags or graphic objects in a computer simulation, the same rules would apply, and that DNA-free explanation would be a fully adequate one [86]. The same could be said of Darwin's model of evolution by Natural Selection, which as well as applying to biological objects could also in theory (and perhaps in fact, although that is disputed) apply to such things as chemical or socio-cultural or software evolution - Richard Dawkins' Universal Darwinism [87]. Even if there were no actual biological objects, the theory would still make logical sense. In a systems biology context, one might derive novel rules concerning a set of properties of a gene-regulatory or metabolic network. These rules might turn out to have logical validity in other contexts and different kinds of network, perhaps even in non-biological networks. Of course in the real biological world, Mendelian and Darwinian and metabolic systems phenomena are instantiated in DNA, cells and organisms, but these laws we use to describe their behaviour are to this extent independent of their substrate, what Paul Davies has called *software laws *rather than *hardware laws *[88]. Reductionism does not so much fail here as appear to be an unnecessary complication. Genes *are *more than just DNA, ushering in the morethanism of explanatory ontological emergence.

Perhaps the attempted inter-theoretic reduction of classical to molecular genetics by Schaffner and Hull was too ambitious, in that it concerned the reduction of an entire discipline, but even within narrower areas results have been mixed. A 1998 survey of the progress of reductionism in biology turned up two cases (muscle contraction and the bacteriorhodopsin receptor) where reduction of molecular biology to the level of chemistry has been nearly achieved to the satisfaction of the investigators [89,90], as well as two other areas (hearing and memory) where considerably less progress had been made [91,92]. It should also be noted that the participants in the 1998 exercise were only describing how they felt their areas of expertise *could be *reduced and what their successes and failures had been. It should also be noted that the reports of successful reductionism were all *below *the molecular biological level, i.e. in the reduction *of *molecular biology to chemistry and not above it, i.e. in the reduction of cellular or organismic biological areas *to *molecular biology. No attempt to apply the full logical reductionist framework in the tradition of the Vienna Circle was attempted, nor is it probable that any practising scientist would have the time or inclination to try. Nevertheless, traditional molecular biologists, those who spend their careers exhaustively analysing the functional and structural properties of one or perhaps a handful of genes and/or proteins, still tend to identify themselves as reductionists, perhaps via the influence of reductionism's advocates among the founding fathers of the field or perhaps because their organismic biologist colleagues have always assured them that they are reductionists (and now systems biologists are doing it too). There may also seem to be something suspicious in the abandonment of reductionism. After all, if one ceases to believe that all science is reducible to physics, this implies that physics is not a completely full description of the world, which opens the door to non-physical entities and inevitably to nightmares of vitalism. One may become a temporary ontological holist when enjoying a pianist's performance, but it is quite another thing to take such attitudes into the laboratory. Reductionism as a vague doctrine exerts a strong hold over those who would never dream of actually applying it to the full extent its creators envisaged. In practise, however, whatever their avowed allegiances, molecular biologists are rarely greedy reductionists. Explanation of cellular phenomena is often attempted at the level of gene action, and occasionally at the level of a single mutation affecting the structure of a single protein, but further reduction of this to the level of quantum chemistry is rarely attempted. Molecular biology's reductions are usually only to the level of macromolecules rather than to the atomic level [32]. For the most part, holist-reductionist controversy does not enter very much into traditional molecular biology except in cases such as human behaviour or cognitive neuroscience where the gap between the subject of study (DNA molecules) and the level to be explained (human behaviour and psychology), is very large. In most areas of molecular biology there has been little question that the study of the expression and structure of individual genes was the quickest way forward, and little appetite for any further reduction beyond that level. Inter-theoretic reduction may be dead but the *biological principle of reduction *[84], a commitment to try to reduce wherever possible, to create as many reductive explanations as possible, even in the absence of complete reduction, lives on.

If that were the end of the story, with vague holism opposed to vague reductionism, then there would be reasonable grounds for setting aside what one of the more entertaining science bloggers recently called "the same old boring stuff about reductionism and holism in biology". However, both a new reductionism and, some would say, a new holism have begun to reveal themselves.

## New Kids on the Block: Neo-Reductionism and Relational Biology

In fact, like the original "New Kids on the Block", these theories are by no means young any more. The new reductionism has its roots in the failure of attempts at inter-theoretic reduction in the early 1970s, and because of this it might be justifiably termed a neo-reductionism (by analogy with neo-conservatism). Faced with problems that appeared to leave an opening for explanatory ontological holism, Kenneth Schaffner first retreated to a form of piecemeal reductionism, the *biological principle of reduction*, advocating a plurality of efforts at different levels: "anatomy, neurology, genetics, and so on, study the living organism in each discipline's own terms" [84]. Even Francis Crick hedged his bets around the same time: "The professional scientist will attack wherever he can.... The point of attack is always a matter of tactics." [56]. This stance lives on in today's pragmatic reductionism [93,94] and in advocates of *inclusive *or *multi-scale *systems biology [11,95,96].

More radically, Schaffner split the reductive process into two kinds: *aggregative*, which is part-to-part or entity-to-entity (Schaffner initially held that "gene = DNA" was one of these) and *interactive*, where the reducing theory does not rigidly predict the relationship of the parts. This latter kind allows an acknowledgement of the arguments such as those given by Philip Kitcher, Paul Davies, Richard Dawkins and Michael Ruse concerning the abstractness, the substrate independence, of much biological theory, without abandoning the deterministic relationship between micro-state and macro-state. This concept was carried forward by Alexander Rosenberg, who has argued that traditional reductionism offered a hostage to fortune in the layered model. For Rosenberg, the failure of inter-theoretic reduction is not a failure of the reductive endeavour itself, but rather of the arbitrary structure of theory relationship that it unwisely attempted to explain. Rosenberg also rejects epistemological antireductionist arguments, for instance the irreducible complexity of large networks described above. Far from being a singulitarian however, Rosenberg regards epistemological antireductionism as being merely trivially true, a fact about scientists and computers rather than science [97]. In the place of the layered model, Rosenberg flattens science to physics and engineering [55,97]. Again this is by no means a completely novel argument, having been previously made by J.J.C. Smart in the 1960s [98]. Smart argued what Rosenberg has called a *provincialist *thesis, that biology is just a province of physics, a field where the laws of physics are applied, but which has no laws of its own. Biologists have scarcely been grateful over the years for this generous offer to relieve them of the burden of inter-theoretic reduction, since it smacks of Rutherford's alleged comment that science is "only physics and stamp-collecting" [99]. This argument is also something of a two-edged sword for reductionists/provincialists since it has also been used by their opponents. For instance, Giora Hon [100] has made the absence of well-formed laws in biology the basis of a call for the rethinking of the whole of biology from first principles. Hon's conception of a new biology is far from being a provincialist one, but rather an *autonomist *one structured with new biotonic laws of the type proposed by Walter Elsasser. The Rutherford-Smart argument may free reductionism from the chains of the layered theory model, but in downgrading biology from science to engineering, it also frees holists from the requirement to recognise that molecular biology has achieved any worthwhile theoretical advances.

Rosenberg fortifies the Rutherford-Smart thesis by stripping the layered model out of Schaffner's interactive reduction, and posits that the facts of biology are *supervenient *on the facts of physics [97]. That is to say that although the argument of the explanatory ontological reductionists, that single biological macro-states do not necessarily have single micro-states, is accepted, two systems with the same micro-state will of necessity have the same macro-state. Just as in interactive reduction, one cannot necessarily predict the macro-state from the micro-state, but the macro-state is nevertheless dependent on it - supervenient to it. Changes to the micro-state may in certain circumstances, change the supervenient macro-state, and if so that change will be predictable. This is a subtle distinction, between prediction of a complete macro-state *ab initio *from a given micro-state and prediction of changes to a macro-state given its micro-state and knowledge of previous changes to the same micro-state in terms of macro-state. The relationship between micro-state and macro-state will never be simple, Rosenberg maintains, because Natural Selection will favour any micro-state that happens to correspond to a macro-state with selective advantage. Traditional reductionism with its layered model of biological hierarchies (nucleotide, gene, organism, species, ecosystem) and their corresponding hierarchies of theory (chemistry, molecular biology, physiology, systematics, ecology) made the assumption that real life was as neat as the model. This could never be the case in an evolving biosphere. Rosenberg's solution is a *Darwinian reductionism*, mapping all macro-states back onto micro-states, but recognizing that those micro-states are chaotic and impossible to analyse. Because natural selection works from highly heterogeneous material towards a smaller number of functional ends, there are an immense variety of physical structures that could be, for instance, a wing. It is therefore pointless trying to reduce the functional structures to molecular states.

Neo-reductionists also maintain that the concept of top-down causation is a mirage. For instance, if a macro-state *M1 *is supervenient on a micro-state *m1*, top-down causation issuing from that macro-state *M1 *has its ultimate cause in the micro-state *m1*. An advocate of top-down causation would argue that once the higher-level property *M1 *has been achieved it is legitimate to say that it has certain effects, or can perform certain functions, for instance it might create a new micro-state *m2*. However, this is at best a shorthand for saying that *m1 *causes *m2 *[101]. The reduction of *M1 *ceases to be a meaningful or worthwhile activity. Here, there is no biological hierarchy and no hierarchy of theories to distress the would-be reductionist. This is the meaning of provincialism.

Although neo-reductionism has had a low profile among systems biologists and biologists in general, a new kind of holism, *Relational Biology*, has attracted attention, mostly among those who are dissatisfied with traditional molecular biology but also sceptical about the explanatory capabilities of modern versions of holism [2,7,18]. Developed over some years by Robert Rosen and a small band of disciples [102,103], relational biology does not dispense with the hierarchy of the Vienna Circle, but rather inverts it. Rosen, based on some earlier similar ideas by Elsasser [104], claimed that physics, by virtue of its application to homogeneous molecular structure is in fact not the fundamental science, but actually a special case. Biology, as the science of the complex, is the lowest level in the layer model. The idea that biology is a special case of physics, i.e. that which applies to a special kind of matter - living things - is in this view actually a residue from vitalism. Rosen proposes that biology should be more about the patterns of relations between things than the things themselves. This idea has been independently developed by many biologists, for instance in Richard Dawkins' Universal Darwinism [87], and can be seen among network theory practitioners in systems biology. Rosen, however, goes somewhat further in arguing that when a system contains a sufficient degree of internal feedback loops, it can achieve a state of self-reference and become non-computable by a Turing machine. Furthermore, even relatively simple networks can be shown to be non-computable. The challenge for modern systems biologists, according to Rosen, is to develop new ways of analysing such network structures.

## Conclusions

Reductionism in biology belongs to the victorious research tradition of mechanistic Darwinism. However, technical inter-theory reduction has proved to be a far more difficult process than its creators envisaged. Reduction may be a valid endeavour, but its lack of general applicability has meant that reduction-*ism *has consequently faltered, and the underdog of holism has emerged from its disreputable origins in vitalism and Lamarckism. It is possible to see the vitalist-Lamarckian-holist and mechanist-Darwinian-reductionist intellectual lineages as competing Kuhnian paradigms [82], but the picture of their interaction is more complex than the basic paradigm shift model. From the 1850s to the 1930s their influences waxed and waned over biology, until the latter was finally triumphant in the 1930s. There was no dramatic paradigm shift, since both had considerable support and all working biologists would have been familiar with them. Neither can be said to have replaced the other, one merely died out as a serious explanatory framework. The difficulty of its experiments and doubtfulness of its theory meant that vitalist-Lamarckian-holism had become a *degenerative research programme *[105]. However, these were not merely competing theories - the choice of one or the other implied profound choices about what kind of experiments could be done and the rules by which they would be interpreted. They were genuinely competing paradigms. Calls for a return to holism in systems biology are not calls for a return to the holism of the 1930s, since that particular holism is no longer tenable. Modern holism, at least in those cases where it is well defined, is part of the current paradigm. The paradigm shift-spotters [1,20,21,23,29,31] in systems biology are therefore at least a little premature in their pronouncements.

The broadening of molecular biology into systems biology has created a situation where researchers have a vague inkling that their underlying philosophy is in need of refurbishment, and holism appears to offer much of what is wanted. Novice holists in systems biology should not feel embarrassed by the vagueness of their convictions; molecular biologists were for many years equally vague about their reductionism. Holism, probably a mixture of the explanatory ontological and epistemological varieties may become the quasi-official philosophy of systems biology with some new Francis Crick taking up the role of chief cheerleader and everybody else just getting on with the lab work. However, given the opportunities for new ideas that arise in the early stages of any new field, this easy adoption of some old philosophy would be a disappointing outcome. There is scope for original thought both within the traditional and more recent concepts of reductionism and holism.

So what *do *we really mean when we speak of holism in systems biology? If systems biologists regard holism as simply the study of larger units, then it is simply a slogan, and it would be best to discard it before it creates divisions where none are necessary. That isn't really holism at all, as it fits perfectly within the traditional hierarchical reductionist scheme. If by holism we mean acknowledgement that irreducible complexity is always going to get in the way of complete understanding and if we are willing to approach that complexity by sacrificing total knowledge in favour of greater approximate understanding, then that is epistemological antireductionism. This may be a new direction but it is within what we might call the current paradigm and certainly does not render traditional molecular biology obsolete. If we go beyond mere complexity to an insistence on an ontological antireductionism that requires the formulation of novel emergent or biotonic laws, then we are still within the realms of the currently comprehensible. After all, as the critics of reductionism have long insisted, biology is full of examples of such things. Whether such laws are non-reducible in principle or merely in practice is a perilous prediction to make. Since reductions are always provisional, the data can surprise the anti-reductionists as much as it can the reductionists.

Outside of the traditional reductionist framework, the neo-reductionism of supervenience theory has so far been relatively unapplied. Given that it purports to escape from the traps of traditional reductionism by scrapping hierarchical models of nature and the corresponding layered models of scientific theory thereby rendering traditional antireductionist critiques meaningless, systems biology would be an ideal testing ground for its cogency. It would be a pity if the challenge were not taken up. If Darwinian reductionism really does transcend the old reductionist-holist dichotomy, systems biology would be the ideal place to demonstrate it. Likewise, we are now in a position to test Rosen's theories about non-computable network structures, and to search for real biological examples of them. Just as experimental programmes were essential to the victories of mechanism in the 1910s and neo-Darwinism in the 1930s, only those theories that immediately suggest relatively easy experiments will be winners.

## Authors' contributions

DG is the sole author of this work.
